# Assessing the association between periodontitis and premature birth: a case-control study

**DOI:** 10.1186/s12884-021-03700-0

**Published:** 2021-03-12

**Authors:** Peace Uwambaye, Cyprien Munyanshongore, Stephen Rulisa, Harlan Shiau, Assuman Nuhu, Michael S. Kerr

**Affiliations:** 1 Department of Preventive & Community Dentistry, University of Rwanda College of Medicine and Health Sciences, School of Dentistry, Kigali, Rwanda; 2 Department of Community Health, University of Rwanda College of Medicine and Health Sciences, School of Public Health, Kigali, Rwanda; 3 Department of Obstetrics & Gynecology, University of Rwanda College of Medicine and Health Sciences, School of Medicine and Pharmacy, Kigali, Rwanda; 4 Department of Periodontology, University of Maryland, College Park, USA; 5 Department of Physiotherapy, University of Rwanda College of Medicine and Health Sciences, School of Health Sciences, Kigali, Rwanda; 6 Arthur Labatt Family School of Nursing, University of Western Ontario, London, Canada

**Keywords:** Periodontitis, Premature birth, Risk factors, Case-control studies

## Abstract

**Background:**

Premature delivery is among the leading causes of perinatal mortality and morbidity in developed societies, which is an important obstetrics problem. Maternal periodontitis is a prevalent condition that has been suspected to be associated with adverse pregnancy outcomes such as premature birth. However, there are still conflicting results about this possible relationship, therefore this study was designed to test the association between maternal periodontitis and premature birth. This study also provides information about a new screening tool recommended for use by nurses and midwives to screen for periodontal diseases during antenatal consultations in order to improve the health of mothers and children.

**Methods:**

A retrospective case-control study was conducted at 12 health facilities in the Southern Province of Rwanda from February to August, 2018. A total of 555 women in the postpartum period were enrolled in the study. Cases and controls were enrolled in a ratio of 1:2; each enrolled case of preterm birth was followed by 2 unmatched control subjects that were next on the register and who delivered at term gestation. A total of 185 cases of preterm deliveries and 370 controls of term delivery were enrolled in the study. Multivariate regression analysis was used and the independent variables were hierarchically entered in three groups: The first group involved demographic variables that were put in the regression model as Step 1. The second group was made up of other potential risk factors that were placed in the regression model as the second step. Periodontitis was entered in the final regression step, as it was hypothesized as the main predictor variable.

**Results:**

A statistically significant association was found between periodontitis and premature birth. Women who had periodontitis had 6 times the odds of giving birth to premature birth infants compared to women who had no periodontitis (OR: 6.360, 95% CI 3.9, 10.4).

**Conclusion:**

The study results indicate that periodontitis is strongly associated with premature birth. Preventive solutions including the use of a periodontitis screening tool for nurses and midwives during antenatal care consultations, are highly recommended.

## Background

Premature births are among the leading causes of perinatal mortality and morbidity globally [1]. In the United States, premature births and associated complications, such as immature lung development, reduced feeding ability and inferior weight gain, result in longer hospital stays and a higher cost of health care, and are responsible for up 70% of prenatal and infant mortality. Almost a quarter of prematurely born infants required hospitalization at least once during their first year, mostly associated with reduced birth weight [2].

Neonatal delivery before 37 weeks of gestation is the leading cause of low birth weight [3]. Premature birth is one of the major healthcare challenges and is associated with many potential consequences like lifelong disabilities and high health care costs [4]. Premature birth is the leading cause of neonatal mortality in low-income countries [4]. Premature deliveries occurred in approximately 12.9 million births worldwide, representing around 9.6% of all births, although there are clear regional discrepancies. Specifically, African nations reported a higher incidence of premature births (18%) followed by the United States (12–13%) and Europe (5–9%). According to a demographic health survey in Rwanda (DHS, 2014–2015), the incidence of low birth weight deliveries was 6.3% [5], and according to the Rwanda Population Reference Bureau, premature birth complications are among the top ten causes of child mortality in Rwanda [6].

Studies have identified numerous risk factors related to premature birth and they can be categorized in three ways: a) non-gynecological causes: low socio-economic status, malnutrition, early maternal age, short maternal stature, low maternal weight, low maternal heart volume, smoking, alcohol consumption, inadequate prenatal care, stress, genitourinary tract infections and malaria, b) gynecological causes: uterine malformations, uterine adhesions and pregnancy with intrauterine infection, c) obstetric causes: primiparity in both young and old mothers, short inter-conception interval, grand multiparity, previous premature births, previous stillbirth, multiple pregnancies, low insertion of the placenta and chorioamnionitis [7].

Although several risk factors are known, 25% of premature births are believed to be of unknown etiology [8]. The recent review of Puertas et al. (2018) documented evidence from several studies examining the possible link between periodontitis with premature birth [8–12]. In 1996, Offenbacher and colleagues described this relationship between maternal periodontitis and premature delivery [8]. The study of Bobetsis et al. [13] argued that, based on biological plausibility, periodontitis can contribute to premature birth through bacteremia where toxins and their products derived from maternal periodontitis can reach the bloodstream and cause injury to the placenta unit and pass into the amniotic fluid, leading to chorio-amniotic infections, increasing the risk of premature birth. It has also been reported that the dissemination of local inflammation throughout the body may contribute to preterm low birth weight [10]^.^ Studies suggest that inflammation in periodontal tissues due to periodontitis increases secretion of different inflammatory cytokines, notably interleukin B (IL-1β), interleukin IL-6, interleukin 8 (IL-8), interleukin 17 (IL-17), and tumor necrosis factors alpha (TNF-α) [8, 10, 14–17].

In Africa, studies have associated poor periodontal status with premature birth and low birth weight deliveries. For example, a study done in Nigeria by Umoh and colleagues concluded that periodontal disease management reduced the prevalence of low birth weight deliveries [18]. The recent review conducted by Jajoo et al. [17], similarly indicated the need for predontitis treatment for reducing the premature births . Although the review highlighted the association between periodontal infection and the risk of premature birth, the literature search conducted did not find any research pointing to periodontal screening during pregnancy. Moreover, in most African countries, such as Rwanda, nurses and midwives are on the frontline of providing antenatal care consultation. Therefore, the current study aims to assess the association between periodontitis and premature birth with the long term goal of initiating a screening tool to be used by nurses and midwives for the screening of periodontitis during antenatal consultations.

## Methods

### Study design and setting

A retrospective unmatched case-control study was conducted to test the association link between periodontitis and premature birth. The study is considered retrospective because both the exposure and the outcome happened prior to subject enrolment. In this study the outcome was preterm delivery and the hypothesized exposure was presence of maternal periodontitis. Participants were selected through hospital registers whereby the research team would verify cases of preterm deliveries and the controls would be selected from the next 2 full-term births on the register. An a priori estimate of the required sample size was calculated using G*Power 3.1.9.6. by Faul [19], Kiel, Germany. The researcher used a small effect size, an alpha of 0.05 and a power of 0.95 with 10 predictors. The estimated sample of cases of 185 was combined with a 1:2 ratio of unmatched controls, totalling 370, thus the total required sample size was 555. The research team completed the exams and questionnaires with study subjects until the required sample was reached. The study was conducted at 12 health facilities in in 6 districts of the Southern Province of Rwanda from February to August 2018, including Kamonyi, Muhanga, Ruhango, Nyanza, Huye and Gisagara. Corresponding district hospitals and nearby health centers were selected for the study as well as a referral hospital in Huye.

### Data sources and measurement

A structured clinical exam and standardized questionnaire were used to collect information regarding the presence of periodontal infection among pregnant women attending antenatal care clinics in the Southern Province of Rwanda. The study adapted the questionnaire from the WHO Oral Health Assessment Tool for Adults of 2013 [20] for use in the Rwandan context. The questionnaire was sent to experts for content validation.

Prior to use in the study in southern Rwnda, the questionnaire was piloted in Nyamata District Hospital in the Eastern Province to ensure that it captured all the required information and that the questions were clear to respondents. After the pilot study, the feedback from the participants was considered and any questions that were not clear were corrected accordingly. The questionnaire was translated in Kinyarwanda using forward and backward translation whereby this questionnaire was first translated in Kinyarwanda from English and then it was again translated back to English by another translator to determine whether the content remained consistent.

The questionnaire collected information on the following variables: age of the respondent, education level, health and lifestyle behaviors (e.g. tobacco use), socio-economic status, mother’s weight, number of previous pregnancies, previous preterm delivery, weight gain during pregnancy, illnesses during pregnancy and stress during pregnancy. The main outcome variable of interest was premature birth.

### Participants and inclusion/exclusion criteria

The inclusion criteria were postpartum mothers aged 18–35 who delivered singleton infants in all selected health facilities within 1 to 5 days before recruitment. Those women who delivered premature were recuited as cases and those who delivered at term were recruited as controls. Maternal registers were checked in order to determine who could be recruited as case or controls based on the above criteria. Cases and controls were enrolled in a ratio of 1:2 and each enrolled case of premature birth was followed by 2 control subjects who delivered at term gestation that were next on the register. A case was defined as any delivery below 37 weeks and a control was defined as any delivery of 37 weeks and above. We used ratio of 1:2 in order to increase the study power and reduce data collection time as there were limited cases of premature birth. Mothers with twin infants, those with systemic conditions like uncontrolled diabetes, HIV infection and those without teeth in one or more sextants were excluded from the study. Women established risk factors for prematurity, like abnormal placentation, eclampsia, uterine abnormalities, and other pregnancy complications that may easily lead to prematurity were also excluded from the study so that they would not bias the results. Other variables that were likely to cause prematurity were documented via the study questionnaire and were controlled during the multivariate analysis.

### Data collection procedures

A periodontal examination was performed on all women enrolled in the study. The study examiners used a Williams graduated periodontal probe to perform the periodontal clinical examination [21]. Six data collectors were calibrated by a qualified dentist on how to perform a periodontal examination to ensure that they all understood it in the same way, to help reduce the chance of reporting different findings for the same patients. The calibration process focused on probe design, gentle and constant force of probing and proper angulations. While no formal agreement analysis was conducted, the examiners were required to examine at least 2 patients and the oral examination training was conducted until all examiners were in agreement with each other and with the trainer. The calibration results was considered valid only when there was no descripancy in results and when the results between the two examiners were consistent. The examinations assessed bleeding on probing, probing depth and clinical attachment loss measured in mm at six different sites on each tooth (buccal-mesial, mid-buccal, buccal-distal, lingual-mesial, mid-lingual and lingual-distal). The study subjects were examined at their bedsides by the lead researcher and calibrated research assistants using a periodontal probe, intraoral mirror and headlights. A gentle probing force was applied to guide the tip into the periodontal pocket until the resistance was felt. The pocket depth was measured using the gingival margin as a reference point. The mothers in the study group were also asked about their dental care practices and their smoking habits. To help prevent bias, the research team members were blind to the study status of the cases and controls.

The current study defined periodontitis as presence of pocket depth greater than 3 mm on either maxilla or mandible or both and presence of interdental clinical attachment loss (CAL) on ether maxilla, mandible or both of 2 mm or above and buccal or oral CAL of 3 mm or above [22].

Based on Jati et al [23], gingival recession was defined as “apical migration of marginal gingiva and characterized by gradual displacement of gingiva away from the cemento-enamel junction that results in the root surface exposure to the oral environment”.

Clinical attachment loss was measured as follows: when the gingival margin was at the cemento-enamel junction and there was no recession, then the CAL was equal to the pocket depth; when the gingival margin was apical to the cemento-enamel junction, CAL was equal to pocket depth plus gingival recession; when the gingival margin was on the anatomical crown in case of gingival overgrowth, CAL was equal to pocket depth minus gingival recession. CAL was not considered in some of the specific cases that were of non-periodontal cause. For example, when the gingival recession was of traumatic origin, dental caries extending in the cervical area of the tooth and in the cases of recession by malposition of the tooth [22].

The current study defined some of the questionnaire variables as follows; i) physical trauma as a wound on the body that was caused by a sudden physical injury, for example, an accident; ii) violence as any behavior or action that intends to hurt someone, physical or verbal and iii) stress during pregnancy as anything that causes emotional strain or tension to the pregnant women.

### Data analysis

IBM SPSS for windows version 21.0 (IBM corp., Armonk, New York, USA) was used for all data analyses [24]. Descriptive statistics, including chi-square analysis was conducted as part of the background to the main hypothesis testing analysis that used multiple logistic regression. The study regression model was built using a three-step modelling approach. Hierarchical multivariate logistic regression analysis was used with the study variables being entered into the analysis using three groups: the demographic variables that were significant in the univariate analysis (age and employment status) were entered first in the regression model as step 1, followed by the second group in step 2, which included other known potential risk factors and confounders in this study such as ever used tobacco, mother’s weight, inter-conception period, whether premature delivery was experienced before, whether stress was experienced during pregnancy, malaria during pregnancy, urinary tract infection, physical trauma and violence during pregnancy. The third and final step of the regression analysis, added periodontitis status to the model, as it was hypothesized as the study’s main exposure variable**.**

The odds ratio was obtained with 95% confidence intervals, thus statistical significance was defined as *p* < 0.05. The fit of the logistic regression models was assessed using regression diagnostic information, such as the − 2 log likelihood and Hosmer-Lemshow chi square values.

## Results

A total of 555 women in the post-partum period were enrolled and screended in the study. Recruitment and data collection continued until the full sample was accumulated. In total, there were 185 cases and 370 controls. The participants were from 6 districts of the Southern Province of Rwanda and from 12 health facilities. The participants were distributed with respect to age, education level, employment status, socio-economic status. The mean age was 27.35 (SD = 5.2) ranging from 18 to 35 years. Most participants (*n* = 338, 60.9%) were in the age range of 26–35 years, 47.6% had primary education, 38.7%, had secondary education, 7% had tertiary education and 6.7% had no formal education. The majority of the study participants (*n* = 513, 92.4%) are from a rural setting, 63.2% are farmers and 55.5% are in category 3 (low) or the “Ubudehe”/ socio-economic status as categorized using the Rwanda national socio-economic status categories. When these demographic variables were associated with premature birth, only employment status was statistically associated with premature birth (*p* = 0.054) (see Table 1).

**Table 1:** Demographic characteristics of participants

Variables	Cases N (%)	Controls, N (%)	Total	Chi-square	*p*-value
Age category					
16–25	68 (12.3)	149 (26.8)	217 (30.1)	0.639	0.24
26–35	117 (21.1)	221 (39.8)	338 (60.9)		
Education level					
No formal eduction	14 (2.5)	23 (4.1)	37 (6.6)	5.864	0.118
Primary level	96 (17.3)	168 (30.3)	264 (47.6)		
Secondary level	68 (12.3)	147 (26.5)	215 (38.7)		
Tertiary education	7 (1.3)	32 (5.8)	39 (7.1)		
Employment status					
Farmer	130 (70.3)	221 (59.7)	351 (63.2)	7.635	0.054
Employed	15 (8.1)	56 (15.1)	71 (12.8)		
Student	9 (4.9)	23 (6.2)	32 (5.8)		
Not employed	31 (16.8)	70 (18.9)	101 (18.2)		
Residence					
Rural	175 (31.5)	338 (60.9)	513 (92.4)	1.855	0.115
Urban	10 (1.8)	32 (5.8)	42 (7.7)		
Social economic					
Category 1	18 (3.2)	38 (6.8)	56 (10.1)	0.4	0.819
Category 2	67 (12.1)	124 (22.3)	191 (34.4)		
Category 3	100 (18.0)	208 (37.5)	308 (55.5)		

### Regression analysis results: factors associated with premature birth among pregnant women attending antenatal care clinics

To assess if there was a relationship between premature birth and the study vaiables, characteristics were compared initially in a univariate fashion between the cases and controls (Table 2). Results from the univariate regression analysis, suggest that 11 variables namely: periodontitis, ever used tobacco, mother’s weight, malaria during pregnancy, urinary tract infection during pregnancy, inter-conception period, previous premature delivery, stress and violence during pregnancy were all possibly associated with premature birth (i.e the *p*-value was less than 0.1) and therefore eligible for the multivariate logistic regression analysis.

**Table 2:** Association between premature birth and potential risk factors

Variable	Cases N (%)	Controls N (%)	Total	Chi-square	*p*-value
Periodontitis					
Healthy	31 (16.8)	229 (61.9)	260 (46.9)	100.9	< 0.001
Periodontitis	154 (83.2)	141 (38.1)	295 (53.2)		
Ever used tobacco					
Yes	5 (2.7)	2 (0.5)	7 (1.3)	4.63	0.031
No	180 (97.3)	368 (99.5)	548 (98.7)		
Mothers’ weight (Kg)					
30–50	37 (20.0)	20 (5.4)	57 (10.3)	29.19	< 0.001
51–75	143 (77.3)	332 (89.7)	475 (85.6)		
76 & above	5 (2.7)	18 (4.9)	23 (4.1)		
History of Malaria					
Yes	56 (30.3)	78 (21.1)	134 (24.1)	5.69	0.017
No	129 (69.7)	292 (78.9)	421 (75.9)		
History of urinary tract infection					
Yes	28 (15.1)	32 (8.7)	60 (10.8)	5.38	0.02
No	157 (84.9)	338 (91.4)	495 (89.2)		
History of respiratory tract infection					
Yes	6 (3.2)	5 (1.4)	11 (2.0)	2.27	0.132
No	179 (96.8)	365 (98.7)	544 (98.0)		
Other illness during pregnancy					
Yes	5 (2.7)	6 (1.6)	11 (2.0)	0.74	0.39
No	180 (97.3)	364 (98.4)	544 (98.0)		
History of premature delivery					
Yes	28 (15.1)	3 (0.8)	31 (5.6)	47.98	< 0.001
No	157 (84.9)	367 (99.2)	524 (94.4)		
History of stress					
Yes	55 (29.7)	57 (15.4)	112 (20.2)	15.71	< 0.001
No	130 (70.3)	313 (84.6)	443 (79.8)		
History of trauma					
Yes	10 (5.4)	12 (3.2)	22 (4.0)	1.514	0.218
No	179 (94.6)	358 (96.8)	533 (96.0)		
Violence during pregnancy					
Yes	31 (16.8)	19 (5.1)	50 (9.0)	20.321	< 0.001
No	154 (83.2)	351 (94.9)	505 (91.0)		
Other causes of stress					
Yes	30 (16.2)	36 (9.7)	66 (11.9)	4.952	0.026
No	155 (83.8)	334 (90.2)	489 (88.9)		
Inter-conception period					
12–24 Months	55 (29.7)	52 (14.1)	107 (19.3)	19.474	< 0.001
Above 24 months	130 (70.3)	318 (86.0)	448 (80.7)		

Multivariate regression analysis was then performed and the variables were hierarchically grouped into three groups: whereby demographic variable of employment status was put first in the regression model as step 1 and followed by other potential factors of ever used tobacco, mother’s weight, last pregnancy, previous premature delivery, history of stress, presence of malaria or urinary tract infection during pregnancy, physical trauma during pregnancy and domestic violence during pregnancy. In the third step of the regression model, periodontitis was added on its own as it was hypothesized as the study’s main exposure variable.

After adjusting for the other variables in the model, the mutiple logistic regression analysis revealed a strong independent association between periodontitis and premature birth, where periodontitis could lead to a six-fold higher risk of delivering premature infants compared to women who had no periodontitis (OR: 6.360, 95% CI 3.9, 10.4). It is worth noting that only previous pre-term birth (OR = 10.6) had a higher risk estimate than periodontitis status. Other significant results from Table 3 suggest that low maternal weight, previous history of prematurity, exposure to violence during pregnancy and a shorter interconception period were also significantly associated with pre-term birth.

**Table 3:** Multivariate logistic regression analysis of premature birth with possible risk factors (Final model)

Variables	Categories	Odds Ratio	***p***-value	95% Confidence Interval
Periodontitis		6.360	< 0.001	3.94–10.27
Employment status	Farmer	1		
	Employed	0.861	0.684	0.42–1.77
	Students	0.694	0.524	0.23–2.14
	Not employed	1.548	0.132	0.88–2.73
Tobacco use	Yes	3.394	0.165	0.60–19.07
Mother’s weight in Kgs	51–75	1		
	30–50	3.207	0.001	1.64–6.28
	≥76	0.322	0.106	0.08–1.27
History of Malaria	Yes	1.521	0.106	0.91–2.53
History of Urinary Tract Infection	Yes	1.539	0.196	0.80–2.96
History of premature delivery	Yes	10.596	< 0.001	2.87–39.15
History of stress during pregnancy	Yes	0.809	0.758	0.21–3.11
History of Violence during pregnancy	Yes	5.382	0.009	1.51–19.15
Other causes of stress	Yes	1.652	0.457	0.44–6.20
Inter-conception period	> 24 months	1		
	12–24 Months	2.168	0.006	1.25–3.76

## Discussion

Premature birth is a major healthcare challenge and is associated with many potential consequences such as lifelong disabilities and high healthcare costs [4]. Premature delivery, which was defined in this study as any delivery within a period of less than 37 weeks, accounts for almost 15 million neonates born prematurely worldwide, mostly in low and middle-income countries [25]. Various risk factors have been identified related to the mother’s premature birth. These maternal risk factors include previous preterm birth, age, weight, socio-economic status, smoking, multiple pregnancies, low prenatal care, nutrition, stress, genito-urinary tract infections, and malaria. In addition, some studies have reported on the possible relationship between periodontitis and premature birth.

Many studies have reported the association between maternal age and premature birth; both extreme ages either under 18 years or above 40 years are considered to be risk factors for premature birth [26–29]. Though the majority of the studies concluded that the older maternal age was associated with higher risk of premature birth, Ambrogio and colleagues in their study observed that both extreme ages that is, maternal age under 17 and advanced age over 40 were independent factors for premature birth (OR 2.97; 95%CI 1.24, 7.14, *P* < 0.005) [26]. This was echoed by Florent and co-authors who also found that both advanced age and lower age were risk factors for premature birth [28].

On the other hand, Mumghamba in Tanzania compared mothers who had PTB infants and those with normal weight infants and reported that PLBW had a lower mean age [30]. Our study shows no association between maternal age and premature birth. In this study, most mothers were young, whereby the mean age was 27.35 (SD 5.2), ranging from 18 to 35 years This limited age range could be the reason why there was no statistical association between maternal age and premature birth in this study. Similarly, the study done in Uganda by Muwazi who also worked on a young population which also showed no association beteeen age and premature birth [31].

Maternal weight has also been reported to be associated with premature birth, whereby in our study the logistic regression revealed that mothers with low weight had higher chances of premature deliveries as opposed to mother with normal weight whereas the odds of having a premature birth baby was 3.2 times for underweight compared to those with normal weight mothers (OR: 3.207 95% CI 1.64, 6.28). Our study findings were echoed by Zhen and colleagues, who revealed that neonates born to underweight mothers had higher chances of preterm delivery (RR 1.21, 95% CI 1.14, 1.25,) [32]. Also in the current study, it was found that the history of violence during pregnancy was significantly associated with premature delivery where by women who experienced violence during pregnancy had 5.3 times higher risk of premature deliveries as opposed to other women who had no history of violence during pregnancy. These findings are in line with the results of the study done in Peru by Sixto Sanchez and colleagues on the risk of spontaneous preterm birth in relation to maternal exposure to intimate partner violence during pregnancy, and results revealed that the women who reported exposure to violence during pregnancy had a 2.1 fold increased risk of preterm birth compared to those who reported no exposure to violence (OR:2.1 95%CI 59,2.68) [33] . The other variables found to be associated with premature birth in this study included: the interval between last pregnancies and the current and previous premature birth.

In this study, the presence or absence of periodontitis disease was a major predictor of preterm delivery. After controlling for other variables, the logistic regression analysis revealed a strong association between periodontitis and premature birth, where periodontitis could lead to a six-fold higher risk of giving birth to premature birth infants compared to women who had no periodontitis (OR = 6.360, 95% CI 3.9, 10.4). It is note worthy that our study results are different in comparison to other studies, for instance, the study done in Germany by Noack and colleagues who did not find any association between periodontitis and premature birth [34]. Similarly, Davenport in 2002, also in a case-control study, did not detect any association between periodontitis and premature birth [35] and also Mumghamba in Tanzania in 2017 did not find any evidence to support a link between periodontitis and premature birth [30].

However, our study findngs are in agreement with several other studies that have supported the association between periodontitis and premature birth. Offenbacher et al., [8] reported for the first time that there was a possible relationship between maternal periodontitis and delivery of a preterm infant where they reported that periodontitis during pregnancy could lead to seven times higher risk of premature birth. Lopez and colleagues also assessed the risk of premature birth and low birth weight in women with periodontitis in the USA and found that pregnant women with periodontitis were at a higher risk of giving birth to premature infants with low birth weight (*p* = 0.0004; RR = 3.5) [36]. Several other studies also confirmed the association [9, 10, 12, 37–40]. A systematic review by Teshome in 2016 concluded there was an association between periodontitis and premature birth [41].

Also, in Africa, other studies have examined the link between premature birth and low birth weight deliveries and these were significantly associated with poor periodontal status; for example, a study done in Nigeria by Umoh and colleagues revealed that periodontal treatment was effective in preventing low birth weight deliveries [18]. In Uganda, Wandera and colleagues also found that mothers with periodontal problems and poor oral hygiene during pregnancy had a greater risk of premature birth as opposed to mothers who did not have periodontitis during pregnancy [42]. Lastly, Muwazi in Uganda in his cross-sectional study reported an association between gingival recession and premature birth [31].

### Policy implications

The study results suggest there is a strong association between maternal periodontitis and the risk of preterm birth. There is a need to improve awareness of non-dental professionals working in antenatal care clinics especially nurses and midwives about the possible obstetric implications of periodontal diseases and to include periodontal screening in the antenatal care package for early detection. The study results indicate there is a need to proactively promote periodontal health during pregnancy.

### Limitations of the study

The limitations of this study include those relevant to most case control studies, including possiblerecall bias. For example, the reported occurrence of some illnesses, like UTI and other medical conditions during pregnancy was based on mothers’ self-report of symptoms and not on laboratory confirmation and therefore over-reporting was possible. Also only mothers who had live births were interviewed and their babies assessed for gestational age. The study did not address factors associated with preterm stillbirth. Lastly, the training and calibration of the data collection examiners was not formalized and no formal agreement analysis was done therefore it is possible that there was still some imprecision of periodontal examination results between examiners.

## Conclusion

The study results suggest that periodontitis is associated with a six-fold increase in the risk of premature birth. Thus, preventive solutions such as having the development of a periodontitis screening tool for nurses and midwives during antenatal care consultations is highly recommended.

## Data Availability

The datasets used and/or analyzed during the current study are available from the corresponding author upon reasonable request.

## Abbreviations

CAL: Clinical Attachment Loss
CI: Confidence Interval
DHS: Demographic Health Survey
IL-6: Interleukin-6
PD: Pocket Depth
SD: Standard Deviation
TNF-α: Tumor Necrosis Factor-alpha
TSAM: Training Support Access Model
WHO: World Health Organization

## References

[1] Chawanpaiboon S, Vogel JP, Moller AB, Lumbiganon P, Petzold M, Hogan D (2019). Global, regional, and national estimates of levels of preterm birth in 2014: a systematic review and modelling analysis. Lancet Glob Health.

[2] Khadem N, Rahmani ME, Sanaei A, Afiat M. Association between preterm and low-birth weight with periodontal disease: a case-control study. Iran J Reprod Med; 2012;10:561–566. Available from: http://www.ncbi.nlm.nih.gov/pubmed/25246927 [cited 2016 Aug 27]. Shahid Sadoughi University of Medical Sciences and Health Services. PMC4169850 25246927

[3] Tielsch JM. Global Incidence of Preterm Birth. Nestle Nutr Inst Workshop Ser; 2015;81:9–15. Available from: http://www.ncbi.nlm.nih.gov/pubmed/26111559 [cited 2018 Apr 12]. Karger Publishers. 10.1159/000365798 26111559

[4] Newnham JP, Kemp MW, White SW, Arrese CA, Hart RJ, Keelan JA. Applying Precision Public Health to Prevent Preterm Birth. Front Public Health 2017;5:66. Available from: http://journal.frontiersin.org/article/10.3389/fpubh.2017.00066/full; [cited 2018 Jun 22]. Frontiers. 10.3389/fpubh.2017.00066 PMC5379772 28421178

[5] National Institute of Statistics of Rwanda (NISR) [Rwanda], Ministry of Health (MOH) [Rwanda], ICF International. Rockville: NISR, MOH, and ICF International; 2015.

[6] Heidi Worley. Population Reference Bureau. Available from: https://www.prb.org/rwanda-maternal-health/. [cited 2018 Jun 22]

[7] Oliveira F, Dutra Oliveira AMS, Cota LOM. Interrelation Between Periodontal Disease and Preterm Birth: Preterm Birth; 2013. 10.5772/54977.

[8] Offenbacher S, Katz V, Fertik G, Collins J, Boyd D, Maynor G, et al. Periodontal Infection as a Possible Risk Factor for Preterm Low Birth Weight. J Periodontol. 1996;67:1103–1113. Available from: http://www.joponline.org/doi/10.1902/jop.1996.67.10s.1103 [cited 2017 Oct 12] 10.1902/jop.1996.67.10s.1103 8910829

[9] Haerian-Ardakani A, Eslami Z, Rashidi-Meibodi F, Haerian A, Dallalnejad P, Shekari M, et al. Relationship between maternal periodontal disease and low birth weight babies. Iran J Reprod Med; 2013;11:625–630. Available from: http://www.ncbi.nlm.nih.gov/pubmed/24639799 [cited 2017 Aug 8]. Shahid Sadoughi University of Medical Sciences and Health Services. PMC3941365 24639799

[10] Bansal M, Khatri M, Kumar A, Bhatia G. Relationship between maternal periodontal status and preterm low birth weight. Rev Obstet Gynecol; 2013;6:135–140. Available from: http://www.ncbi.nlm.nih.gov/pubmed/24826203 [cited 2017 May 20]. MedReviews, LLC. PMC4002189 24826203

[11] Walia M, Saini N. Relationship between periodontal diseases and preterm birth: Recent epidemiological and biological data. Int J Appl Basic Med Res; 2015;5:2–6. Available from: http://www.ncbi.nlm.nih.gov/pubmed/25664259 [cited 2016 Aug 27]. Medknow Publications. 10.4103/2229-516X.149217 PMC4318095 25664259

[12] Puertas A, Magan-Fernandez A, Blanc V, Revelles L, O’Valle F, Pozo E, et al. Association of periodontitis with preterm birth and low birth weight: a comprehensive review. J Matern Neonatal Med. 2018;31(5):597–602. 10.1080/14767058.2017.1293023 28282773

[13] Bobetsis YA, Graziani F, Gürsoy M, Madianos PN. Periodontal disease and adverse pregnancy outcomes. Periodontol 2000; 83:154–174. Available from: https://onlinelibrary.wiley.com/doi/abs/10.1111/prd.12294 [cited 2020 Aug 5]. Blackwell Munksgaard. 10.1111/prd.12294 32385871

[14] Jeffcoat MK, Geurs NC, Reddy MS, Cliver SP, Goldenberg RL, Hauth JC. Periodontal infection and preterm birth: results of a prospective study. J Am Dent Assoc. 2001;132:875–880. Available from: http://www.ncbi.nlm.nih.gov/pubmed/11480640 [cited 2019 Jan 22]. 10.14219/jada.archive.2001.0299 11480640

[15] Africa CWJ (2011). Oral colonization of gram-negative anaerobes as a risk factor for preterm birth. Virulence..

[16] El-Gharib MN, Nassar MM, Elabyary MT, Elhawary TM, Elshourbagy SH (2010). Link between periodontal Diseases, inflammatory markers and preterm low birth weight infants. Libr Acad.

[17] Jajoo NS, Shelke AU, Bajaj RS, Patil PP, Patil MA. Association of periodontitis with pre term low birth weight – A review. Placenta. 2020;95:62–8. 10.1016/j.placenta.2020.03.006 32452403

[18] Umoh A, Ojehanon P, Savage K. Effect of maternal periodontal status on birth weight. Eur J Gen Dent; 2013;2:158. Available from: http://www.ejgd.org/text.asp?2013/2/2/158/112318 [cited 2016 Aug 29]. Medknow Publications and Media Pvt. Ltd.

[19] Faul F, Erdfelder E, Lang AG, Buchner A (2007). G*power 3: a flexible statistical power analysis program for the social, behavioral, and biomedical sciences. Behav Res Methods.

[20] Petersen PE, Ramon J. B. Oral Health Questionnaire for Adults. Oral Heal Surv Basic Methods. 2013:111–5 Available from: http://apps.who.int/iris/bitstream/handle/10665/97035/9789241548649_eng.pdf?sequence=1.

[21] Periodontal probes. Available from: https://www.slideshare.net/malvika014/periodontal-probes-64860621. [cited 2021 Feb 23]

[22] Tonetti MS, Greenwell H, Kornman KS. Staging and grading of periodontitis: Framework and proposal of a new classification and case definition. J Clin Periodontol. 2018;45(20):S145–61. 10.1111/jcpe.13152. 10.1111/jcpe.12945 29926495

[23] Jati AS, Furquim LZ, Consolaro A (2016). Gingival recession: its causes and types, and the importance of orthodontic treatment. Dental Press J Orthod.

[24] IBM Corp. Released 2012. IBM SPSS statistics for windows, version 21.0.Armonk, Ny: IBM Corp Available from: https://www.ibm.com/support/pages/how-cite-ibm-spss-statistics-or-earlier-versions-spss

[25] Quinn J-A, Munoz FM, Gonik B, Frau L, Cutland C, Mallett-Moore T, et al. Preterm birth: Case definition &amp; guidelines for data collection, analysis, and presentation of immunisation safety data. Vaccine; 2016;34:6047–6056. Available from: http://www.ncbi.nlm.nih.gov/pubmed/27743648 [cited 2018 Apr 12]. Elsevier. 10.1016/j.vaccine.2016.03.045 PMC5139808 27743648

[26] Londero AP, Rossetti E, Pittini C, Cagnacci A, Driul L. Maternal age and the risk of adverse pregnancy outcomes: a retrospective cohort study. BMC Pregnancy Childbirth. 2019;19:261. Available from: https://bmcpregnancychildbirth.biomedcentral.com/articles/10.1186/s12884-019-2400-x [cited 2020 Jan 31] 10.1186/s12884-019-2400-x PMC6651936 31337350

[27] Soltani M, Tabatabaee HR, Saeidinejat S, Eslahi M, Yaghoobi H, Mazloumi E, et al. Assessing the risk factors before pregnancy of preterm births in Iran: a population-based case-control study. BMC Pregnancy Childbirth. 2019;19:57. Available from: https://bmcpregnancychildbirth.biomedcentral.com/articles/10.1186/s12884-019-2183-0 [cited 2020 Jan 31] 10.1186/s12884-019-2183-0 PMC6364407 30727983

[28] Fuchs F, Monet B, Ducruet T, Chaillet N, Audibert F. Effect of maternal age on the risk of preterm birth: a large cohort study. PLoS One. 2018;13 Public Library of Science. 10.1371/journal.pone.0191002 PMC5791955 29385154

[29] Wagura PM (2014). Prevalence and factors associated with preterm birth at.

[30] Mumghamba EGS, Manji KP. Maternal oral health status and preterm low birth weight at Muhimbili National Hospital, Tanzania: a case-control study. BMC Oral Health; 2007;7:8. Available from: http://www.ncbi.nlm.nih.gov/pubmed/17594498 [cited 2017 May 27]. BioMed Central. 10.1186/1472-6831-7-8 PMC1924845 17594498

[31] Muwazi L, Rwenyonyi CM, Nkamba M, Kutesa A, Kagawa M, Mugyenyi G, et al. Periodontal conditions, low birth weight and preterm birth among postpartum mothers in two tertiary health facilities in Uganda. BMC Oral Health; 2014;14:42. Available from: http://www.ncbi.nlm.nih.gov/pubmed/24773772. [cited 2018 Dec 6] BioMed Central. 10.1186/1472-6831-14-42 PMC4022576 24773772

[32] Han Z, Mulla S, Beyene J, Liao G, Mcdonald SD. Maternal underweight and the risk of preterm birth and low birth weight: a systematic review and meta-analyses. Int J Epidemiol. 2011. p. 65–101. 10.1093/ije/dyq195 21097954

[33] Liu C, Långström N, Ekéus C, Frisell T, Cnattingius S, Hjern A. Paternal violent criminality and preterm birth: a Swedish national cohort study. BMC Pregnancy Childbirth. 2020:1–9. 10.1186/s12884-020-02964-2 PMC7238610 32429861

[34] Noack B, Klingenberg J, Weigelt J, Hoffmann T. Periodontal status and preterm low birth weight: a case control study. J Periodontal Res; 2005;40:339–345. Available from: http://doi.wiley.com/10.1111/j.1600-0765.2005.00808.x [cited 2018 Apr 12]. Wiley/Blackwell 10.1111/j.1600-0765.2005.00808.x 15966912

[35] Davenport ES, Williams CECS, Sterne JAC, Murad S, Sivapathasundram V, Curtis MA. Maternal Periodontal Disease and Preterm Low Birthweight: Case-Control Study. J Dent Res; 2002;81:313–318. Available from: http://journals.sagepub.com/doi/10.1177/154405910208100505 [cited 2018 Apr 7]. SAGE Publications. 10.1177/154405910208100505 12097443

[36] López NJ, Smith PC, Gutierrez J. Higher Risk of Preterm Birth and Low Birth Weight in Women with Periodontal Disease. J Dent Res; 2002;81:58–63. Available from: http://journals.sagepub.com/doi/10.1177/002203450208100113 [cited 2017 Oct 12]. SAGE PublicationsSage CA: Los Angeles, CA 10.1177/002203450208100113 11820369

[37] Marakoglu I, Gursoy UK, Marakoglu K, Cakmak H, Ataoglu T. Periodontitis as a risk factor for preterm low birth weight. Yonsei Med J; 2008;49:200–203. Available from: http://www.ncbi.nlm.nih.gov/pubmed/18452254 [cited 2019 Feb 25]. Yonsei University College of Medicine. 10.3349/ymj.2008.49.2.200 PMC2615330 18452254

[38] Reza Karimi M, Hamissi JH, Naeini SR, Karimi M. The Relationship Between Maternal Periodontal Status of and Preterm and Low Birth Weight Infants in Iran: A Case Control Study. Global J Health Sci; 2015;8:184–188. Available from: http://www.ncbi.nlm.nih.gov/pubmed/26652090 [cited 2019 Feb 25]. Canadian Center of Science and Education. 10.5539/gjhs.v8n5p184 PMC4877198 26652090

[39] Lohana MH, Suragimath G, Patange RP, Varma S, Zope SA. A prospective cohort study to assess and correlate the maternal periodontal status with their pregnancy outcome. J Obstet Gynecol India; 2017;67:27–32. Available from: http://link.springer.com/10.1007/s13224-016-0920-0. Springer India 10.1007/s13224-016-0920-0 PMC5306100 28242964

[40] Rakoto-Alson S, Tenenbaum H, Davideau J-L. Periodontal Diseases, Preterm Births, and Low Birth Weight: Findings From a Homogeneous Cohort of Women in Madagascar. J Periodontol. 2010;81:205–213. Available from: http://www.ncbi.nlm.nih.gov/pubmed/20151798 [cited 2018 Jun 23]. 10.1902/jop.2009.090351 20151798

[41] Teshome A, Yitayeh A. Relationship between periodontal disease and preterm low birth weight: systematic review. Pan Afr Med J; 2016;24:215. Available from: http://www.ncbi.nlm.nih.gov/pubmed/27800070 [cited 2018 Apr 12]. African Field Epidemiology Network. 10.11604/pamj.2016.24.215.8727 PMC5075444 27800070

[42] Wandera M, Astrøm AN, Okullo I, Tumwine JK. Determinants of periodontal health in pregnant women and association with infants’ anthropometric status: a prospective cohort study from Eastern Uganda. BMC Pregnancy Childbirth; 2012;12:90. Available from: http://www.ncbi.nlm.nih.gov/pubmed/22950749 [cited 2016 Aug 29]. BioMed Central. 10.1186/1471-2393-12-90 PMC3515345 22950749

